# The abundance and host-seeking behavior of culicine species (Diptera: Culicidae) and *Anopheles sinensis *in Yongcheng city, people's Republic of China

**DOI:** 10.1186/1756-3305-4-221

**Published:** 2011-11-24

**Authors:** Xiao-Bo Liu, Qi-Yong Liu, Yu-Hong Guo, Jing-Yi Jiang, Dong-Sheng Ren, Guang-Chao Zhou, Can-Jun Zheng, Yan Zhang, Jing-Li Liu, Zhi-Fang Li, Yun Chen, Hong-Sheng Li, Lindsay C Morton, Hua-Zhong Li, Qun Li, Wei-Dong Gu

**Affiliations:** 1 State Key Laboratory for Infectious Disease Prevention and Control, China CDC Key Laboratory of Surveillance and Early-Warning on Infectious Disease, National Institute for Communicable Disease Control and Prevention, Chinese Center for Disease Control and Prevention, Beijing, 102206, China; 2 Yongcheng Center for Disease Control and Prevention, Yongcheng, 476600, China; 3 University of South Florida, College of Public Health, 13201 Bruce B. Downs Blvd., MDC 54, Tampa, FL 33412; 4 Office for Disease Control and Emergency Response, Chinese Center for Disease Control and Prevention, Beijing, 102206, China; 5 Department of Medicine, William C. Gorgas Center for Geographic Medicine, University of Alabama at Birmingham, Birmingham AL.35294, USA; 6 EDEB/NCEZID/CDC, Atlanta, Georgia 30329, USA

**Keywords:** Host-seeking behavior, mosquito, culicine species, *Anopheles *vectors, ecology, malaria elimination

## Abstract

**Background:**

The knowledge of mosquito species diversity and the level of anthropophily exhibited by each species in a region are of great importance to the integrated vector control. Culicine species are the primary vectors of Japanese encephalitis (JE) virus and filariasis in China. *Anopheles sinensis *plays a major role in the maintenance of *Plasmodium vivax *malaria transmission in China. The goal of this study was to compare the abundance and host-seeking behavior of culicine species and *An. sinensis *in Yongcheng city, a representative region of *P. vivax *malaria. Specifically, we wished to determine the relative attractiveness of different animal baits versus human bait to culicine species and *An. sinensis*.

**Results:**

*Culex tritaeniorhynchus *was the most prevalent mosquito species and *An. sinensis *was the sole potential vector of *P. vivax *malaria in Yongcheng city. There were significant differences (P < 0.01) in the abundance of both *An. sinensis *and *Cx. tritaeniorhynchus *collected in distinct baited traps. The relative attractiveness of animal versus human bait was similar towards both *An. sinensis *and *Cx. tritaeniorhynchus*. The ranking derived from the mean number of mosquitoes per bait indicated that pigs, goats and calves frequently attracted more mosquitoes than the other hosts tested (dogs, humans, and chickens). These trends were similar across all capture nights at three distinct villages. The human blood index (HBI) of female *An. sinensis *was 2.94% when computed with mixed meals while 3.70% computed with only the single meal. 19:00~21:00 was the primary peak of host-seeking female *An. sinensis *while 4:00~5:00 was the smaller peak at night. There was significant correlation between the density of female *An. sinensis *and the average relative humidity (P < 0.05) in Wangshanzhuang village.

**Conclusions:**

Pigs, goats and calves were more attractive to *An. sinensis *and *Cx. tritaeniorhynchus *than dogs, humans, and chickens. Female *An. sinensis *host-seeking activity mainly occurred from 19:00 to 21:00. Thus, we propose that future vector control against *An. sinensis *and *Cx. tritaeniorhynchus *in the areas along the Huang-Huai River of central China should target the interface of human activity with domestic animals and adopt before human hosts go to bed at night.

## Background

Malaria remains a serious global public health threat and causes substantial morbidity and mortality in the world. The current estimate of human lives at risk from *Plasmodium vivax *malaria is 2.6 billion [1,2] and South and East Asia account for 52% of the total *P. vivax *malaria burden [3]. The estimated global cost of *P. vivax *malaria, including lost productivity, cost of health care, and transport to clinics, is between U.S. $1.4 and $4 billion per year [3]. The interruption of *P. vivax *malaria transmission worldwide is still one of the greatest challenges for international health and development communities [4]. Despite significant reductions in the overall burden of malaria in the 20^th ^century [5-7], this parasitic disease still represents a major public health problem in China [6,8], with dramatic re-emergence in the Huang-Huai River region of central China in 2001 [5]. In Henan Province [9-13] and Anhui Province [14,15], large-scale epidemics in recent years have caused a major public health concern. This situation not only has an impact on the region's economic development and people's living standards, but also poses a challenge for the routine malaria control strategy. In response to the global initiative to eradicate malaria [16,17], an action plan for malaria elimination was proposed by the Chinese Ministry of Health in 2009 and a national elimination campaign was launched by the Chinese Government in 2010, to eliminate malaria in most endemic regions by 2015 and to achieve ultimate national elimination by 2020 [18].

*Anopheles sinensis*, *Anopheles lesteri *[19,20], *Anopheles minimus *and *Anopheles dirus *are considered to be four important vector species of malaria in China [21,22]. However, malaria outbreaks and re-emergences were only in areas with *An. sinensis *in recent years [5]. In studies conducted in the areas along the Huang and Huaihe River, *An. sinensis *plays an important role in the maintenance of *P. vivax *malaria transmission [23,24]. Yongcheng city, as a re-emergence region of *P. vivax *malaria in Henan Province, is one of the major malaria epidemic areas [25]. After extensive control efforts, malaria was nearly eliminated in the 1990s [9,25]; however, epidemics resurged and maintained in the area in the 2000s with the highest cases of 2,890 in 2006 [25,26]. According to surveillance data of Yongcheng Center for Disease Control and Prevention (Yongcheng CDC), a decline in the malaria incidence rate was observed [27]. Based on a study conducted by Zhou et al in 2010, *An. sinensis *was considered to be the sole potential vector of *P. vivax *malaria in Yongcheng city [5,25,27]. The estimated vectorial capacity of *An. sinensis *population in this area was 2.78 times higher in 2010 (0.4983) than in the 1990s [28]. The epidemiological consequences of this change are unknown, but this observed entomological change cannot be ignored when transitioning from malaria control to elimination in China. In recent years, several factors, such as the adjustment of regional agricultural structures and the reduction of biological barriers (calf, pig, etc.), are likely to increase human-vector contact and increase the transmission rate of *P. vivax *malaria [5].

Vector abundance, host-seeking behavior and preference [29] are all important components of disease-transmission cycles [29,30]. Host-seeking behavior has been operationally defined as the in-flight orientation of the avid female toward a potential blood meal host [31]. Host-seeking typically commenced shortly after sunset and usually peaks during the succeeding one to three hours, the hottest and driest period at night [32,33]. The host-seeking strategies used by arthropod vectors can, in part, affect the efficiency of disease transmission [30,34]. Mosquitoes which prefer to feed on animals are less important in transmitting human disease than those which prefer to feed on humans [35]. In particular, the knowledge of vector abundance and host-seeking activities is of critical importance for integrated vector control. *Culex tritaeniorhynchus *is the primary vector of the Japanese encephalitis (JE) virus [36,37], and *Culex pipiens pallens *is the primary vector of the JE virus and filariasis in China. The host-seeking behavior of culicine species is of great significance in the epidemiology of the JE virus and filariasis and the host-seeking behavior of *Anopheline *mosquitoes is of great significance in the epidemiology of malaria [38,39]. The host-seeking behavior can be studied by a variety of methods [40,41], such as human landing catch (HLC) [42], human bait indoors and outdoors [38,43], indoor residual spray (IRS), baited mosquito nets, baited resting boxes [44], light traps [45,46], etc. Several approaches including serologic techniques, enzyme-linked immunosorbent assay (ELISA) and DNA-based techniques, have been applied to study the blood-feeding behavior of mosquitoes, black flies, ticks and other blood-feeding arthropods as it relates to host-parasite interactions and pathogen transmission [47-58].

Since culicine species and *An. sinensis *play a distinct role in disease transmission, the abundance and host-seeking behavior of culicine species and *An. sinensis *are of fundamental epidemiological importance [38], however, little information is available regarding species composition, abundance, host-seeking behavior, and the level of anthropophily exhibited by each species in Yongcheng city. The goals of this study were to compare the abundance and host-seeking behavior of culicine species and *An. sinensis *in villages of Yongcheng city characterized by different levels of historical incidence of *P. vivax *malaria and determine the relative attractiveness of different animal baits versus human bait to culicine species and *An. sinensis*. Such information is required in order to understand the comprehensive vector biology of culicine species and *An. sinensis*, for the design of effective vector control strategies and for implementation of the ongoing malaria elimination campaign in China.

## Methods

### Study site

**Figure 1:**
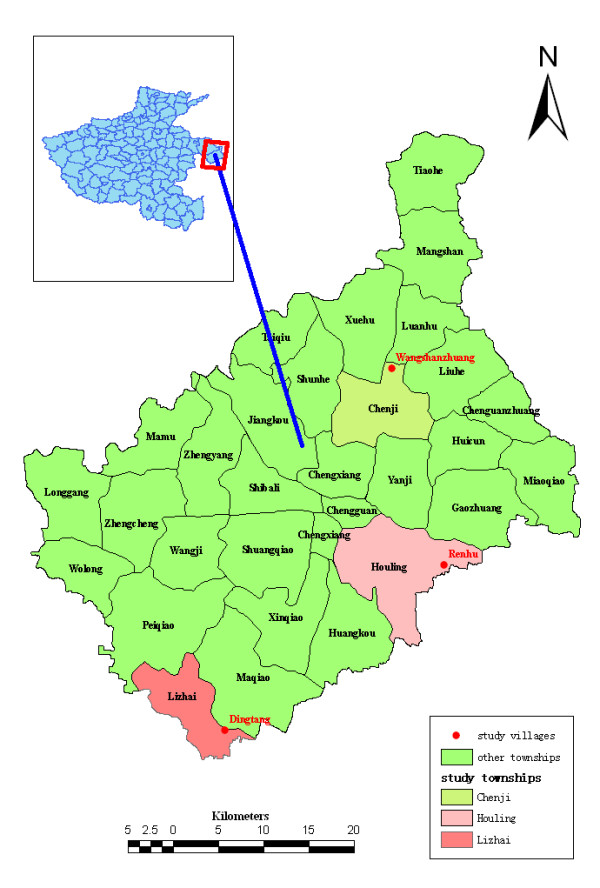
**Map showing the study villages in Yongcheng city of Henan Province, People's Republic of China**. Red dot represents the study village; Green square frame represents the boundary of Yongcheng city; Yellow square frame represents the low risk township, Chenji; A little pink square frame represents the intermediate risk township, Houling; Pink square frame represents the high risk township, Lizhai.

The current study was conducted in three villages of Yongcheng city characterized by different levels of historical incidence of *P. vivax *malaria. These included a high risk village Dingtang (Lizhai township, annual average incidence rate > 100/100,000), an intermediate risk village Renhu (Houling township, annual average incidence rate 10~100/100,000 and a low risk village Wangshanzhuang (Chenji township, annual average incidence rate < 10/100,000) [25], (Figure 1). The inhabitants of these villages live in houses which are made with bricks. These villages are located in an area between latitudes of 33°42' and 34°18', and longitudes of 115°58' and 116°39'. The Tuohe River is the main environmental feature in the area. The river breaks into small ponds during the hot and dry season, and ponds which are formed by the river become adequate breeding habitats of *An. sinensis *and culicine species. Most of the regions are plain at 33 meters altitude. The climate is warm temperate from May to October, and the annual average temperature is 14.3°C. The range of annual rainfall is between 556.2 mm and 1,648.9 mm, and mostly rainfall is concentrated to June, July, August, and September. The main crops of these villages are wheat, soybean, corn, and a small amount of cotton and potato. During summer, most of local residents tend to sleep outdoors [21].

Besides the difference in the levels of historical incidence of *P. vivax *malaria, other characteristic differences between the three studied villages are as follows: First, Dingtang village is adjacent to Guoyang County, and Renhu village is adjacent to Suixi County. Guoyang and Suixi County are unstable regions of *P. vivax *malaria in Anhui Province while Wangshanzhuang village is not adjacent to Anhui Province. Second, the water-body distributions and appropriate breeding habitats of culicine species and *An. sinensis *larvae in Dingtang village and Renhu village are more than those in Wangshanzhuang village. During the study period, there were many ponds and canals that contained endogenous lotus, and a large number of *Anopheline *mosquitoes and culicine species larvae were observed in Renhu village and Dingtang village. Third, the number of animal hosts in Renhu village and Dingtang village was larger than that of Wangshanzhuang village. Based on the investigative data of the village doctors, Dingtang village covers approximately 3,500 acres of arable land, with a population of 1,890 people, 1 calf, and 150 goats within 480 households, Renhu village covers approximately 4,500 acres of arable land, with a population of 1,875 people, 60 calves, 70 pigs, and 180 goats within 470 households, Wangshanzhuang village covers approximately 1,600 acres of arable land, with a population of 750 people, and 5 calves, 45 pigs, and 70 goats within 170 households, respectively.

### Host-seeking behavior experiments

Host-seeking behavior experiments of culicine species and *An. sinensis *were conducted from August 28^th ^to October 5^th^. The reason that these experiments were conducted during this period is that this period was considered to be the epidemic episode of *P. vivax *malaria along the Huaihe River region in China [59]. Three villages were sampled in different weeks because of limited man power and material resources. Outdoor bednet traps [34,60] were employed to study the host-seeking activity of culicine species and *An. sinensis*. The size of the bednet traps was 3.6 m × 2.4 m × 1.6 m. There was twenty centimeters between the floor and the bottom of the bednet traps. The locations of the bednet traps were close to ponds, ditches and drains, in a line or ellipse, at intervals of 50 m. The distance from the bednet traps to the nearest resident's house was about 60 m, and people living in the range of this distance near to the water-bodies had higher risk of malaria infection than those living beyond the distance [5]. In each sampled village, major local domestic animals, such as calves, pigs, goats, dogs, and chickens, were used as animal baits to attract mosquitoes to the bednet traps. Some members of staff at Yongcheng CDC and China CDC were selected as human baits. These members (under double bednet traps to avoid mosquitoes bites) were also used repeatedly throughout the entire duration of the study to avoid bias [35,45].

To control for the plausible confounding bias of locations of the bednet traps and collection nights, one 5 × 5 Latin square design and two 6 × 6 Latin square design experiments [34,46,61] were conducted. The Latin square design is used where researchers desire to control the variation in an experiment that is related to rows and columns in the field. Treatments (baits) were assigned at random within rows (nights) and columns (locations of the bednet trap), with each treatment once per row and once per column [34]. During this period, three independent experiments were conducted, with one experiment per village. A 5 × 5 Latin square design experiment was conducted in Dingtang village because five types of bait, namely calves, goats, dogs, chickens and humans, existed in this village while two 6 × 6 Latin square design experiments were conducted in Renhu village and Wangshanzhuang village because six types of bait, namely calves, pigs, goats, dogs, chickens, and humans existed in these two villages.

**Figure 2:**
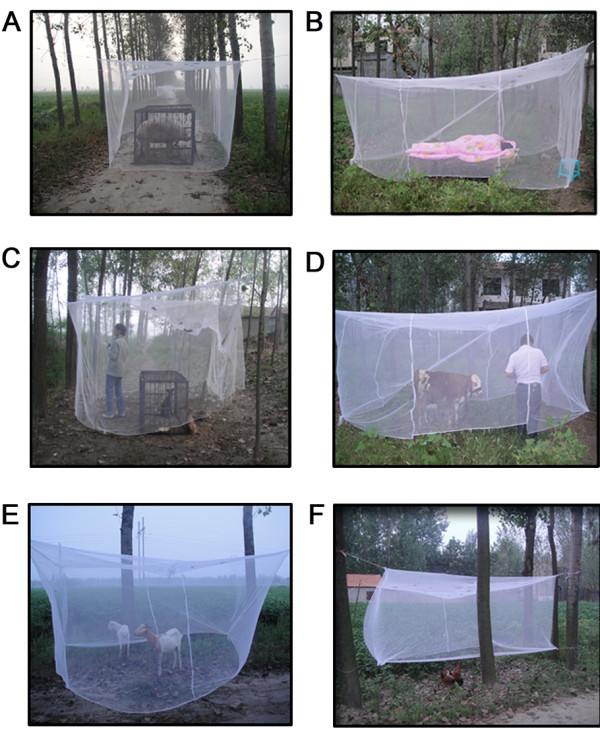
**Map showing the photos of bednet traps in the field in Yongcheng city of Henan Province, People's Republic of China**. Photo A represents pig-baited bednet trap; Photo B represents human-baited bednet trap; Photo C represents dog-baited bednet trap; Photo D represents calf-baited bednet trap; Photo E represents goat-baited bednet trap; Photo F represents chicken-baited bednet trap.

Before the experiment began, the bednet traps were fixed at previously selected locations. The locations of the bednet traps were alternated every night, each bednet trap being moved with the bait to avoid the effect of odor contamination. Calves, pigs, dogs, goats, and chickens were placed into the bednet traps. Dogs and pigs were put into metal cages under the bednet traps to prevent staff from being bitten by them (Figure 2). Metal cages were welded in Yongcheng city and the bednet traps were fixed before 19:00. The interval between 19:00 and 7:00 was decided upon by consulting the literature, which reported the host-seeking behavior of culicine species and *An. sinensis *[61-63]. Calves and pigs were chosen as young small animals and experiments were carried out with one calf, one pig, two goats, two dogs, and five chickens at a time under the bednet traps [34]. Every hour, all mosquitoes inside the bednet traps were collected by an electrical aspirator for 15 minutes per hour throughout the 12 hour period [64]. Collection time periods were subdivided into early evening (19:00~22:00), late evening (22:00~01:00), post-midnight (01:00~ 04:00), and pre-dawn (04:00~07:00) [64]. The staff was divided into four teams with three people in each team. The first team worked for the first three hours from 19:00 to 22:00, followed by the second team from 22:00 to 01:00. The third team worked from 01:00 to 04:00, and the last team worked from 04:00 to 07:00.

Data was recorded for collection dates and sites, sequences of locations of the bednet traps, collection nights, and numbers of each mosquito species. Temperature (°C) and relative humidity (%) were the average value from a weather web in China http://www.weather.com.cn. Ambient outdoor air temperature and relative humidity of each collection hour was recorded using a WS-1 Thermo-Hygrometer device. Since some events such as heavy rains would prevent host-seeking activity, mosquito sampling occasions were scheduled based on the pattern of weather conditions. During the trapping days, however, rainfall values (mean minimum and maximum in mm) were obtained from a local meteorological station located in Yongcheng city, so that the effect of this environmental factor could be evaluated while controlling for between-night variation in mosquito abundance.

### Blood meal identification

Collected mosquitoes were narcotized by ether and morphologically identified in the field using commonly accepted guidelines [65,66]. All collected *Anopheline *mosquitoes were put into 1.5 ml centrifuge tubes. All blood-fed female *An. sinensis *were put individually into tubes and then transported to the laboratory of The Department of Vector Biology and Control in China CDC. To reveal the species of *Anopheline *mosquitoes in Yongcheng city [5,66], a ribosomal DNA PCR assay was conducted [67-72]. Fifty percent of blood-fed female *Anopheline *mosquitoes which were selected as equal proportions from different bednet traps in three villages were further identified to species. A Qia Amp DNA Mini Kit (Qiagen Inc., CA) was used and DNA was extracted from the thorax of *An. sinensis *according to the manufacturer's instructions. The PCR conditions used were identical to a study of Ma et al in China [68]. A multiplex PCR assay was conducted to identify the origins of blood meal from the same *Anopheline *mosquitoes used for species identification. The whole blood of domestic animals and humans was obtained in Yongcheng city. Blood DNA of blood-fed female *An. sinensis *was extracted from the mosquitoes' abdomen and the multiplex PCR conditions and primers used in the current study were the same as that previously described by Kent & Norris in 2005 [52] (Table 1).

**Table 1:** Primer sequences for the cytochrome b-based polymerase chain reaction blood meal identification assay

^**1**^**Primers**	5'-3'sequences	Product size with Unrev1025
Human741F	GGC TTA CTT CTC TTC ATT CTC TCC T	334
Cow121F	CAT CGG CAC AAA TTT AGT CG	561
Pig573F	CCT CGC AGC CGT ACA TCT C	453
Goat894F	CCT AAT CTT AGT ACT TGT ACC CTT CCT C	132
Dog368F	GGA ATT GTA CTA TTA TTC GCA ACC AT	680
Unrev1025	GGT TGT CCT CCA ATT CAT GTT A	-

^1^Primers of this multiplex PCR assay were designed based on the sequence differences in cytochrome b sequences.

### Data analyses

The species composition of mosquitoes in the three villages was calculated. Abundance (mean catch per bait per night) of culicine species and *An. sinensis *were calculated for each site and bait. The number of culicine species and *An. sinensis *of each bait was log (x+1) transformed and subjected to variance analysis after a satisfactory check for normality of the distribution. Univariate analysis of variance (general linear models functions) was used to test the main effect and interaction effects of bait types, collection nights, and locations of the bednet traps. Probabilities of the Univariate analysis of variance were at α = 0.05 level. Pair wise comparison had been performed to rank host-seeking behavior of culicine species and *An. sinensis*. Normal distributions of the *An. sinensis *density, the average temperature, and the average relative humidity were tested by One-sample of Kolmogorove-Smirnov Test before using Pearson correlation analysis. The association between the density of *An. sinensis *and the average temperature, between the density of *An. sinensis *and the average relative humidity were examined by Pearson correlation analysis. Data was entered and analyzed by SPSS (Statistical Package for the Social Sciences) statistical software (version 11.5).

## Ethical approval

Ethical approval for this study was obtained from the Ethical Committee of China CDC and permission was also obtained from the Municipal Government, the Municipal Health Bureau and CDC in Yongcheng city.

## Results

### Species composition

A total of 35,312 mosquitoes were captured in the three villages during the study period, of which 76.6% (n = 27,048) were *Cx. tritaeniorhynchus*, 12.2% (n = 4,313) were *Cx. pipiens pallens*, 10.6% (n = 3,755) were *An. sinensis*, 0.02% (n = 9) were *Aedes albopictus*, 0.5% (n = 165) were *Armigeres subalbatus (Coquillett)*, and 0.1% (n = 22) were others. *Cx. tritaeniorhynchus *was the most prevalent mosquito species, and *An. sinensis *was the sole potential vector of *P. vivax *malaria in Yongcheng city. As far as sex proportions of captured *An. sinensis *is concerned, 1.25% (n = 47) out of 3, 755 *An. sinensis *were males, while 98.75% (n = 3,708) were females. Fifty percent (n = 102) of blood-fed female *Anopheline *mosquitoes, which were sampled, about the same proportions from the bednet traps in the three villages, were further identified to species by ribosomal DNA PCR assay. All collected adult female *Anopheline *mosquitoes examined belonged to *An. sinensis*, confirming that there was not any other species of *Anopheline *mosquitoes of Hyrcanus Complex in Yongcheng city [72,73].

### The abundance of female culicine species and *An. sinensis *per baited trap at each study site

The abundance of female culicine species and *An. sinensis *per baited trap at each study site was calculated (Tables 2 &3). There were significant differences in the abundance of female *An. sinensis *for each bait [F (5, 1153) = 53.722, P < 0.01]. There were significant differences in the abundance of female *Cx. tritaeniorhynchus *for each bait [F (5, 1153) = 28.261, P < 0.01]. There were no differences in the abundance of female *Cx. pipiens pallens *for each bait [F (5, 1153) = 1.111, P = 0.353]. Pair wise comparison showed that the rank of abundance of host-seeking female *An. sinensis *for each bait from high to low was pigs (7.27 ± 11.146 catch per bait per night), goats (5.14 ± 8.614 catch per bait per night), calves (4.81 ± 7.553 catch per bait per night), dogs (1.62 ± 4.802 catch per bait per night), humans (0.90 ± 2.364 catch per bait per night), and chickens (0.65 ± 1.422 catch per bait per night). The rank of abundance of host-seeking female *Cx. tritaeniorhynchus *for each bait from high to low was pigs (49.83 ± 115.660 catch per bait per night), goats (31.03 ± 55.729 catch per bait per night), calves (19.84 ± 45.657 catch per bait per night), dogs (16.93 ± 34.208 catch per bait per night), humans (6.19 ± 11.381 catch per bait per night), and chickens (6.18 ± 17.569 catch per bait per night). These findings suggested that there was a consistent trend of the rank of abundance of host-seeking female *An. sinensis *and *Cx. tritaeniorhynchus*.

**Table 2:** ^1^The abundance of female culicine species and *An. sinensis *in the study sites of Yongcheng city, Henan Province, China

^**2**^**The study sites**	The species of mosquitoes	^**3**^**N**	Mean	Std. Deviation	Std. Error	95% Confidence Interval for Mean	
						
						Lower Bound	Upper Bound
Dingtang village	*An. sinensis*	407	1.38	2.72	0.16	1.07	1.69
	*Cx. tritaeniorhynchus*	7,631	25.87	48.79	2.84	20.28	31.46
	*Cx. pipiens pallens*	131	0.44	2.22	0.13	0.19	0.70
Renhu village	*An. sinensis*	2,960	6.85	10.10	0.49	5.90	7.81
	*Cx. tritaeniorhynchus*	14,019	32.45	77.32	3.72	25.14	39.76
	*Cx. pipiens pallens*	2,782	6.44	20.95	1.01	4.46	8.42
Wangshan- zhuang village	*An. sinensis*	341	0.79	1.96	0.09	0.60	0.97
	*Cx. tritaeniorhynchus*	1,801	4.17	11.43	0.55	3.09	5.25
	*Cx. pipiens pallens*	292	0.68	1.29	0.06	0.55	0.80

^1 ^The abundance refers to mean catch per bait per night.

^2 ^Dingtang village in Lizhai township belongs to high levels of historical incidence of *P. vivax *malaria; Renhu village in Houling township belongs to intermediate levels of historical incidence of *P. vivax *malaria; Wangshanzhuang village in Chenji township belongs to low levels of historical incidence of *P. vivax *malaria.

^3 ^N represents the total number of female mosquitoes in the study sites.

**Table 3:** ^1^The abundance of female culicine species and *An. sinensis *in bednet traps baited with different animal and human hosts in Yongcheng city, Henan Province, China

Bait types	The species of mosquitoes	^**2**^**N**	Mean	Std. Deviation	Std. Error	95% Confidence Interval for Mean	
						
						Lower Bound	Upper Bound
Human	*An. sinensis*	182	0.90	2.364	0.166	0.57	1.22
	*Cx.tritaeniorhynchus*	1,256	6.19	11.381	0.799	4.61	7.76
	*Cx. pipiens pallens*	315	1.55	3.315	0.233	1.09	2.01
Calf	*An. sinensis*	977	4.81	7.553	0.530	3.77	5.86
	*Cx.tritaeniorhynchus*	4,028	19.84	45.657	3.205	13.52	26.16
	*Cx. pipiens pallens*	318	1.57	4.732	0.332	0.91	2.22
Goat	*An. sinensis*	1,043	5.14	8.614	0.605	3.95	6.33
	*Cx.tritaeniorhynchus*	6,300	31.03	55.729	3.911	23.32	38.75
	*Cx. pipiens pallens*	576	2.84	12.787	0.897	1.07	4.61
Pig	*An. sinensis*	1,047	7.27	11.146	0.929	5.43	9.11
	*Cx.tritaeniorhynchus*	7,175	49.83	115.660	9.638	30.77	68.88
	*Cx. pipiens pallens*	919	6.38	29.694	2.475	1.49	11.27
Dog	*An. sinensis*	328	1.62	4.802	0.337	0.95	2.28
	*Cx.tritaeniorhynchus*	3,437	16.93	34.208	2.401	12.20	21.67
	*Cx. pipiens pallens*	622	3.06	10.302	0.723	1.64	4.49
Chicken	*An. sinensis*	131	0.65	1.422	0.100	0.45	0.84
	*Cx.tritaeniorhynchus*	1,255	6.18	17.569	1.233	3.75	8.61
	*Cx. pipiens pallens*	455	2.24	7.279	0.511	1.23	3.25

^1 ^The abundance refers to mean catch per bait per night.

^2 ^N represents the total number of female mosquitoes of distinct types of bait.

### Host-seeking activity of female culicine species and *An. sinensis*

As to the host-seeking activity of female *Cx. tritaeniorhynchus*, the results of Univariate analysis of variance demonstrated that there were significant differences among the number of female *Cx. tritaeniorhynchus *captured at each bait [F (5,1105) = 8.117, P < 0.01], and at each bednet location [F(5,1105) = 3.413, P = 0.005], but no differences at each night [F(5,1105) = 1.648, P = 0.145]. There were no interactions between nights and locations of the bednet traps [F (3, 1105) = 0.031, P = 0.993], locations of the bednet traps and baits [F (5, 1105) = 1.782, P = 0.114], and there were interactions between nights and baits [F (3, 1105) = 2.233, P = 0.08]. As far as the host-seeking activity of female *Cx. pipiens pallens *is concerned, there were significant differences among the number of female *Cx. pipiens pallens *captured at each bait [F(5,1105) = 6.989, P < 0.01], at each night [F(5,1105) = 7.118, P < 0.01], and at each bednet location [F(5,1105) = 3.257, P = 0.006]. There were interactions between nights and locations of the bednet traps [F (3, 1105) = 4.170, P = 0.006], nights and baits [F (3, 1105) = 3.903, P = 0.009], and locations of the bednet traps and baits [F (5, 1105) = 3.015, P = 0.01]. There were significant differences among the number of female *An. sinensis *captured at each bait [F (5, 1105) = 13.161, P < 0.01], at each night [F (5, 1105) = 4.460, P < 0.01], but no differences of each bednet location [F (5, 1105) = 0.259, P = 0.90]. There were no interactions between nights and locations of the bednet traps [F (3, 1105) = 0.912, P = 0.434], nights and baits [F (3, 1105) = 1.961, P = 0.118], and locations of the bednet traps and baits [F (5, 1105) = 1.094, P = 0.362].

### Multiple blood meals of female engorged *An. sinensis *highlighted by blood meal analysis

**Figure 3:**
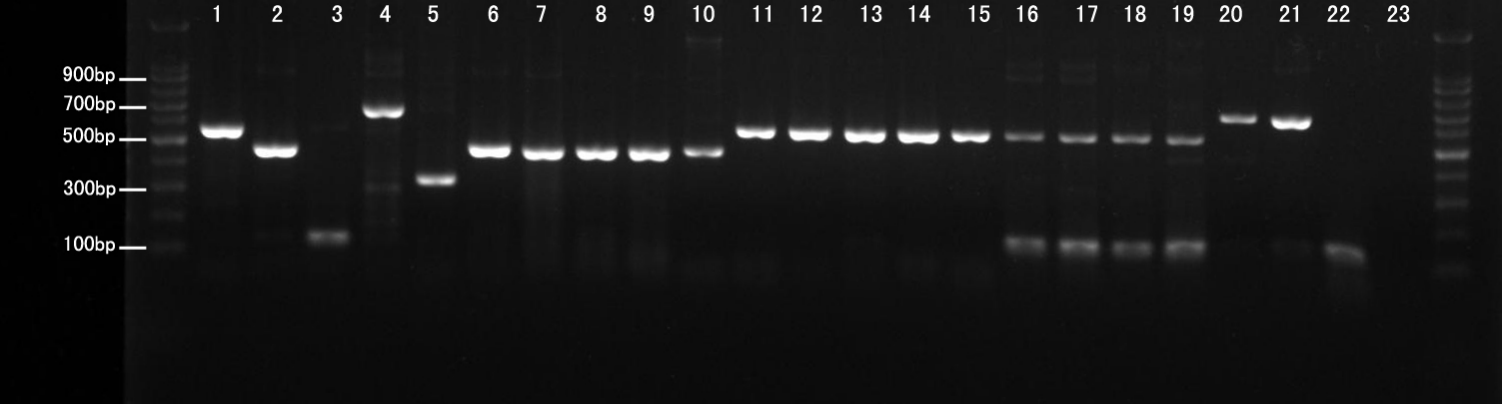
**Ethidium bromide-stained agarose gel showing host-specific cytochrome b polymerase chain reaction products amplified from whole blood DNA extractions and engorged female *An. sinensis *in the field**. Control products amplified from whole blood are shown in lane 1 (calf, 561 bp), lane 2 (pig, 453 bp), lane 3 (goat, 132 bp), lane 4 (dog, 680 bp), and lane 5 (human, 334 bp), respectively. Engorged female *An. sinensis *in three villages in Yongcheng city are shown in lane 6-22, respectively. Lane 23, negative control. Outside lanes are 100 bp DNA ladders. bp = basepairs.

We used a host-specific multiplex PCR assay based on the mitochondrial cytochrome b gene to identify the mammalian blood meals of field-collected blood-fed female *An. sinensis*. This PCR assay correctly identified the blood meals of control groups of blood of different domestic animals 100% of the time and yielded no amplification of unfed *An. sinensis *template DNA. Ethidium bromide-stained agarose gel showed host-specific cytochrome b PCR products amplified from whole blood DNA extractions and collected female blood-fed *An. sinensis *in the field (Figure 3). No blood-fed female *An. sinensis *were collected in chicken-baited mosquito nets during this period. Therefore, only four types of the most common domestic animals in the study sites and humans were included in this multiplex PCR assay. A total of 102 samples of blood-fed female *An. sinensis *were sampled from three villages in Yongcheng city. The result showed that 81 blood meals belonged to a single origin, including pigs (n = 24), calves (n = 23), goats (n = 16), dogs (n = 4), humans (n = 3), and others (n = 11) while 21 blood meals belonged to mixed origin, including calf/goat (n = 5), calf/pig (n = 9), pig/dog (n = 1), calf/pig/goat (n = 2), calf/pig/dog (n = 2), and others (n = 2). The HBI calculated from identification of blood meals of blood-fed *An. sinensis *including mixed meals was 2.94% and 3.70% when computed with only the single meals (Table 4). High proportions of mixed meals were encountered (25.9%).

**Table 4:** Origin of blood meal of engorged female *An. sinensis *in the study sites during the work period

^**1**^**Origin of blood meal of engorged female** *An.sinensis*	The research sites															Total
		
	Dingtang village					Renhu village					Wangshanzhuang village					
		
	Human bednet trap	Calf bednet trap	Goat bednet trap	^2^Pig bednet trap	Dog bednet trap	Human bednet trap	Calf bednet trap	Goat bednet trap	Pig bednet trap	Dog bednet trap	Human bednet trap	Calf bednet trap	Goat bednet trap	Pig bednet trap	^3^Dog bednet trap	
**Single host**																0
Human	3															3
Goat	2		9			1		4								16
Dog					2					2						4
Pig	2	1							12	1				8		24
Calf	1	7				1	7	1			2	3	1			23
Other	3						1		1	2	1	3				11
**Mixed host**																0
Calf/goat			1					4								5
Calf/pig	2											6	1			9
Pig/dog					1											1
Calf/pig/dog					2											2
Calf/pig/goat	1						1									2
Other	1								1							2
Total	15	8	10	0	5	2	9	9	14	5	3	12	2	8	0	102

^1^Numbers of single and mixed blood meals in Yongcheng city. ^2^No pig was kept by the residents of Dingtang village. ^3^No blood-fed *An. sinensis *was captured from dog-baited bednet trap in Wangshanzhuang village. No blood-fed *An. sinensis *were captured from chicken-baited bednet trap.

### Hourly host-seeking activity of female *An. sinensis*

There were significant differences among the average number of captured female *An. sinensis *at different hours at night [F (11, 3697) = 32.542, P < 0.01] (Table 5). Host-seeking activity of female *An. sinensis *showed a similar bimodal pattern at all study sites. The primary peak of female *An. sinensis *was from 19:00 to 21:00 while the smaller peak was from 4:00 to 5:00.

**Table 5:** Variations in the hourly catches of host-seeking female *An. sinensis *in bednet traps baited with different animal and human hosts during 17 nights in Yongcheng city, Henan Province, China

Hour interval	^**1**^**N**	^**2**^**Mean**	Std. Deviation	Std. Error	95% Confidence Interval for Mean	
					
					Lower Bound	Upper Bound
19:00~20:00	426	23.58	21.074	1.021	21.58	25.59
20:00~21:00	494	26.98	20.735	0.933	25.14	28.81
21:00~22:00	307	18.50	14.275	0.815	16.90	20.11
22:00~23:00	286	19.55	19.555	1.156	17.27	21.82
23:00~0:00	255	11.68	10.689	0.669	10.36	13.00
0:00~1:00	218	9.61	6.513	0.441	8.74	10.47
1:00~2:00	296	16.79	11.468	0.667	15.48	18.10
2:00~3:00	311	16.38	11.034	0.626	15.15	17.61
3:00~4:00	255	15.77	13.583	0.851	14.09	17.44
4:00~5:00	315	20.20	13.693	0.772	18.68	21.72
5:00~6:00	232	14.86	12.238	0.803	13.28	16.45
6:00~7:00	313	16.97	12.395	0.701	15.60	18.35
Total	3,708	18.59	16.091	0.264	18.07	19.11

^1^N represents the total number of female mosquitoes of each hour. ^2^The mean of captured mosquitoes refers to the average number of *An. sinensis *per net per hour.

### Variation in the density of female *An. sinensis *with environment variables

Table 6 represents the density of female *An. sinensis *relative to the average temperature and the average relative humidity in the study sites. Each day's collection (12 hours capture) represents the total number of female *An. sinensis *captured in three sites and showed the variation in the density of female *An. sinensis *according to the study period. Pearson's correlation analysis showed that there were no correlations between the density of female *An. sinensis *and the average temperature (r = -0.038, P = 0.951) or the average relative humidity (r = -0.041, P = 0.947) in Dintang village. There were no correlations between the density of female *An. sinensis *and the average temperature (r = -0.658, P = 0.155) or the average relative humidity (r = -0.598, P = 0.210) in Renhu village. There were no correlations between the density of female *An. sinensis *and the average temperature (r = 0.610, P = 0.199), but significant correlations between the density of female *An. sinensis *and the average relative humidity (r = 0. 859, P = 0.029) in Wangshanzhuang village.

**Table 6:** The distribution of the *An. sinensis *density, the average temperature, and the average relative humidity in the study sites

^**1**^**The study sites**	Parameters	^**3**^**N**	Mean	Std.Deviation	Minimum	Maximum	One-Sample Kolmogorov-Simirnov Test	
							
							Kolmogorov-Simirnov Z	**^4^Sig**.
Dingtang village	^2^*The An. sinensis density*(mosquito/net/night)	5	16.28	7.25	7.00	24.40	0.482	0.974
	The average temperature(°C)	5	26.62	3.68	23.00	31.50	0.531	0.940
	The average relative humidity (%)	5	84.20	9.70	71.00	95.00	0.422	0.994
Renhu village	*The An. sinensis density*(mosquito/net/night)	6	82.22	27.13	34.67	111.67	0.490	0.970
	The average temperature(°C)	6	23.63	3.42	19.50	28.50	0.475	0.978
	The average relative humidity (%)	6	78.83	11.80	60.00	90.00	0.488	0.971
Wangshan-zhuang village	*The An. sinensis density*(mosquito/net/night)	6	9.47	9.67	2.50	28.50	0.932	0.351
	The average temperature(°C)	6	17.88	2.77	13.80	21.00	0.449	0.988
	The average relative humidity (%)	6	84.17	6.77	74.00	95.00	0.555	0.918

^1^Dingtang village in Lizhai township belongs to high levels of historical incidence of *P. vivax *malaria; Renhu village in Houling township belongs to intermediate levels of historical incidence of *P. vivax *malaria; Wangshanzhuang village in Chenji township belongs to low levels of historical incidence of *P. vivax *malaria. ^2^The density of *An. sinensis *refers to a total of female *An. sinensis *per net per night. ^3^N refers to the total of night. ^4^Sig. = significance.

## Discussion

The results obtained from the current research demonstrated that the species composition of mosquitoes in Yongcheng city includes *Cx. tritaeniorhynchus*, *Cx. pipiens pallens*, *An. sinensis*, *Ae. albopictus*, and *Ar. subalbatus(Coquillett)*. This was the first time that the species composition of mosquitoes was reported in Yongcheng city. Though *Cx. tritaeniorhynchus*, *Cx. pipiens pallens*, *Ae. albopictus*, and *Ar. subalbatus(Coquillett) *are not vectors of *P. vivax *malaria, these species could be considered as the major nuisance on the basis of their high proportions. The influence of culicine species, especially *Cx. tritaeniorhynchus *and *Cx. pipiens pallens *should not be ignored, since *Cx. tritaeniorhynchus *is the primary vector of JE virus, and *Cx. pipiens pallens *is the primary vector of JE virus and filariasis in China [36]. The ribosomal DNA PCR assay revealed that *An. sinensis *was the sole species of Hyrcanus Complex in identified female *Anopheline *mosquitoes in the study sites. The results of this study were consistent with those reported earlier in China [5,25,27]. Based on previous researches and results of the current PCR assay, it was probable that there weren't any other species of *Anopheline *mosquitoes of Hyrcanus Complex except for *An. sinensis *present in Yongcheng city.

Mosquito host-seeking behavior can be studied by different methods [30,33,74-77]. In the northeastern USA, host-seeking activities and avian host preferences of mosquitoes associated with West Nile virus (WNV) transmission were studied by a custom-designed trap baited with dry ice [77]. To study potential WNV vectors, horse- and bird-baited traps and HLC methods were carried out weekly from May to October 2004 at two Camargue sites [45]. In the Toledo District, Belize, Central America, host-feeding preferences of *Anopheline *species were collected by manual aspiration, mechanical aspiration, and a vehicle-mounted trap [78]. In the present study, baited bednet traps [34,79] were adopted to study the host-seeking behavior of culicine species and *An. sinensis*. The current study was similar to a study in Bernalillo County, New Mexico. Hosts used in their study were calves, chickens, dogs, and horses [80]. The rationale for using baited bednet traps in the current study was that they would separate the zoophilic and the anthropophilic species while other methods of collection, such as HLC and IRS may not be suitable to use for this purpose.

Latin square design (LSD) was employed in the present study because the experimental set-up consisted of several baits tested on 5-6 experimental nights. Previous studies had established that the LSD would greatly reduce potential bias related to the location and alternated position of the baited-bednet samplers, allowing comparison of the attractiveness of different hosts to the mosquitoes in the field [34,46].

The results of variance analysis demonstrated that there were significant differences among the abundance of *Cx. tritaeniorhynchus *for each bait, the abundance of *An. sinensis *for each bait, but no differences of female *Cx. pipiens pallens *for each bait. There were some major characteristics, which might influence the abundance of female *Cx. tritaeniorhynchus *and *An. sinensis *in these villages. First, during the study period, the locations of the bednet traps of Renhu village were near to a wide canal and many ponds (harboring endogenous lotus). High density of culicine and *Anopheline *mosquito larvae were observed from these breeding sites. In another experiment concerning breeding sites of *Anopheline *mosquitoes in 2010, Renhu village had more breeding sites of *Anopheline *mosquitoes than the other two villages. Second, the number of animal baits from high to low was consistent with the abundance of collected female culicine species and *An. sinensis *in the study sites. Differences in the availability of blood meals for female culicine species and female *An. sinensis *might exist within the study villages. Third, the locations selected for our study were sampled at different periods. This could possible explain the observed differences in the number of both *Culicine *and *Anopheline *mosquitoes caught between the villages. Fourth, the local selective pressures within which mosquitoes thrive might be added to the variability of the results.

By far, field studies have revealed that each mosquito species had its own host preference [81,82]. Pair wise comparison showed that the rank of abundance of host-seeking female *Cx. tritaeniorhynchus *and *An. sinensis *for each bait from high to low was pigs, goats, calves, dogs, humans, and chickens, respectively. There was a consistent trend of the rank of abundance of host-seeking activity between female *Cx. tritaeniorhynchus *and *An. sinensis*. In Thailand, *An. sinensis *was almost entirely zoophilic in comparative biting tests involving man and cow; almost none of them were attracted to man [83]. This phenomenon could be explained by an opportunistic feeding behavior present in these species. The anti-vector behavior at the community level, namely practices often used by local people for protection from mosquitoes, also had some influence on the host-seeking behavior of these species. Olfaction may be the major sensory modality involved in the resource searching behavior of insects [84]. In mosquitoes, it is mainly exploited in host-seeking and finding a suitable place for oviposition. Differences in host-preference in the current study were likely to be reflected in their response to different host odors offered [85-88]. However, the focus on the current research was only one aspect of behavior in mosquitoes and cannot fully explain the dramatic re-emergence of malaria in the Huang-Huai River region of central China. Zhou et al [5] suggested that the spatial distribution between malaria cases and water-body, the changing of meteorological factors, and the increasing vectorial capacity and basic reproductive rate of *An. sinensis *were the possible determinants of malaria re-emergence in these areas.

In the current study, successful amplification was obtained by template DNA from the whole blood and female blood-fed *An. sinensis *in the research villages. All bednet traps sampled approximately the same proportions of female blood-fed *An. sinensis*. The results of multiplex PCR assay showed that only 3 blood meals were human in origin, while most of blood meals originated from domestic animals, such as pigs, calves, and goats. The overall HBI calculated from this study including mixed blood meals was 2.94% and 3.70% when compared with only the single blood meal. Similar studies were reported in Japan in 1951 [89]; in north Kyonggi-do in 1962 (1.7%), in 1999 (0.7%), and in 2000 (0.8%) [90]. Though the HBI of these tests was very low, they readily fed on humans in high numbers where domestic animals were not nearby for feeding [91]. The HBI of the current study was higher than that of these findings mentioned above. Gonotrophic discordance, or taking multiple blood meals during the gonotrophic cycle, has been reported in many different mosquito taxa. In these studies, approximately 10% to 40% of blood-fed field specimens contained multiple blood meals [92-99]. Amplification of template derived from mosquito abdomens containing mixed blood meals confirmed that multiple blood meals from different mammals could be detected in a single mosquito. Our studies demonstrated that high proportions of mixed blood meals were encountered (21/102), and cryptic blood meals were likely to be more numerous. Two to three sources of blood meal were detected from the abdomen of female blood-fed *An. sinensis*, and this phenomenon could be explained by a modification of the traditional view of the gonotrophic cycle [34]. This host-feeding behavior can influence pathogen transmission through increased frequency of vector-human contact, or possibly reduce vector-human contact if some blood meals are taken from alternative mammalian hosts [52]. In the current study, we failed to identify the origin of the blood meal in 11 out 102 field specimens examined. Insufficiency in host DNA concentrations due to the low blood meal volume in some mosquito specimens is presumed to be the cause of this failure. In addition, the process of blood digestion may have denatured host DNA, or the mosquito may have fed on an animal not included in the diagnostic assay [52].

The current study demonstrated that the host-seeking activity of female *An. sinensis *showed two peaks. The primary host-seeking activity occurred between 19:00 and 21:00 and a smaller peak between 4:00 and 5:00. It reported that *An. sinensis *bites man and animals soon after dark and throughout the night, with a peak generally in the first quarter of the night [21]. In east China, its peak was from 19:00 to 21:00 [100]. Chow (1949) found that the peak of *An. sinensis *biting buffalo in Chongqing was from 20:00 to 22:00. Zhou et al [5] reported that the biting time of *An. sinensis *was from 19:30 to 24:00 locally, with a peak time from 21:00 to 24:00 in Yongcheng city. The primary peak time in the present study was earlier than that of Zhou' s report [5] and consistent with a previous study in east China [100]. This pattern suggested that vector abatement directed at adult *An. sinensis *would be most effective if initiated from 19:00 to 21:00, namely before human host go to bed at night.

Previous studies have shown that meteorological factors, such as the average temperature, rainfall, and relative humidity have a certain correlation to mosquito density in the corresponding period [101-105]. Temperature was found to be the most important environmental factor, followed by rainfall and relative humidity in the Delphi evaluation. However, relative humidity was found to be more important than rainfall and temperature in the ranking list according to the three single environmental factor regression models [106]. Paaijmans et al [107] believed that the incubation period for malaria parasites within the mosquito is exquisitely temperature-sensitive, and temperature is a major determinant of malaria risk. Yang et al [98] found that the distribution of the mosquito vector was mainly related to relative humidity, which determined the extent of malaria transmission. In the current study, there were no correlations between the density of female *An. sinensis *and the average temperature in the study sites, while significant correlations between the density of female *An. sinensis *and the average relative humidity (r = 0.859, P = 0.029) were observed in Wangshanzhuang village. It could be explained due to the average relative humidity affecting the distribution and breeding of *An. sinensis *[106].

Care needs to be taken in interpreting the results of this study. Several factors may have affected the variability in the abundance and host-seeking behaviour of the culicine mosquitoes and *An. sinensis *in our study. First, biases inherent in the trapping method may have affected the observed number and species composition of mosquitoes within baited traps. Some mosquitoes may fly out from the gaps under the baited bednet traps more easily than others, resulting in some species being underrepresented. Second, the physiological status and infected proportions of mosquitoes were not recorded in the field; therefore, the importance of gravid female *An. sinensis *is not emphasized in the current report. Third, the abundance of *Ae. albopictus*, *Ar. subalbatus (Coquillett)*, and other mosquito species in each site were not calculated because they were poorly represented in our samples. Further research should be undertaken using this multiplex PCR protocol for the identification of the origin of blood meals of female blood-fed *An. sinensis*, in addition, further studies are needed to better characterize the malaria vector and its respective role in malaria transmission. An olfactometry assay could be conducted in the future to clarify if behavior of *An. sinensis *is not directional and possibly provide an indication of how this species will behave in different circumstances. The origin of blood meals of *Cx. tritaeniorhynchus *could also be studied in the future because of its high proportions and epidemiologic implications [108].

## Conclusions

Given the observed data in the studied research sites, the epidemiologic implications of culicine species, especially *Cx. tritaeniorhynchus*, could not be ignored on the basis of their high proportions. *An. sinensis *was the sole potential vector of *P. vivax *malaria in Yongcheng city. Pigs, goats, and calves had a greater tendency to attract both female *Cx. tritaeniorhynchus *and female *An. sinensis *than dogs, humans, and chickens at night. High proportions of mixed meals of female engorged *An. sinensis *were encountered, and cryptic meals were likely to be more numerous. When it comes to the host-seeking activity of female *An. sinensis*, 19:00~21:00 was the primary peak while 4:00~5:00 was the relatively smaller peak at night. These aspects mentioned above are of great importance to the understanding of vector biology of culicine species and *An. sinensis*, and the design of effective vector control strategies. These findings should be considered in the implementation of malaria elimination campaigns in China, especially in malaria re-emergence areas.

## List of abbreviations used

PCR: polymerase chain reaction; JE: Japanese encephalitis; HLC: human landing catch; IRS: indoor residual spray; HBI: human blood index.

## Competing interests

The authors declare that they have no competing interests.

## Authors' contributions

LXB and LQY designed and carried out statistical analysis and wrote the paper. GYH, RDS, ZY, LZF and LJL performed data management. JJY, ZGC and LHS were involved in the execution of the research. LQY, LXB, LM, GYH, GWD drafted and wrote up the manuscript. LQY supervised data collection. LQY, GYH, RDS, ZCJ, LHZ and LQ reviewed this paper for publication. All authors approved the final version of the manuscript.

## References

[B1] Guerra CA Snow RW Hay SI Mapping the global extent of malaria in 2005 Trends Parasitol 2006 22 8 353 358 10.1016/j.pt.2006.06.006 16798089 PMC3111076

[B2] Guerra CA Snow RW Hay SI Defining the global spatial limits of malaria transmission in 2005 Adv Parasitol 2006 62 157 179 16647970 10.1016/S0065-308X(05)62005-2 PMC3145102

[B3] Price RN Tjitra E Guerra CA Yeung S White NJ Anstey NM Vivax malaria: neglected and not benign Am J Trop Med Hyg 2007 77 6 Suppl 79 87 18165478 PMC2653940

[B4] Alonso PL Brown G Arevalo-Herrera M Binka F Chitnis C Collins F Doumbo OK Greenwood B Hall BF Levine MM A research agenda to underpin malaria eradication PLoS Med 8 1 e1000406 10.1371/journal.pmed.1000406 PMC3026687 21311579

[B5] Zhou SS Huang F Wang JJ Zhang SS Su YP Tang LH Geographical, meteorological and vectorial factors related to malaria re-emergence in Huang-Huai River of central China Malar J 2010 9 337 10.1186/1475-2875-9-337 21092326 PMC3003275

[B6] Tang LH Progress in malaria control in China Chin Med J (Engl) 2000 113 1 89 92 11775219

[B7] Zhou ZJ The malaria situation in the People's Republic of China Bull World Health Organ 1981 59 6 931 936 6978199 PMC2396122

[B8] Lin H Lu L Tian L Zhou S Wu H Bi Y Ho SC Liu Q Spatial and temporal distribution of falciparum malaria in China Malar J 2009 8 130 10.1186/1475-2875-8-130 19523209 PMC2700130

[B9] Sleigh AC Liu XL Jackson S Li P Shang LY Resurgence of vivax malaria in Henan Province, China Bull World Health Organ 1998 76 3 265 270 9744246 PMC2305715

[B10] Xu BL Su YP Shang LY Zhang HW Malaria control in Henan Province, People's Republic of China Am J Trop Med Hyg 2006 74 4 564 567 16606984

[B11] Liu XZ Xu BL Malaria situation and evaluation on the control effect in Henan Province during 1990-2005 Chinese Journal of Parasitology and Parasitic Diseases 2006 24 3 226 229 17094630

[B12] Zizhao L Luoyuan S Lian Z Dongfang L Yunpu S Control strategies of malaria in Henan Province, China Southeast Asian J Trop Med Public Health 1999 30 2 240 242 10774685

[B13] Liu X Jackson S Song J Sleigh AC Malaria control and fever management in Henan Province, China, 1992 Trop Med Int Health 1996 1 1 112 116 10.1046/j.1365-3156.1996.d01-5.x 8673815

[B14] Wang LP Fang LQ Xu X Wang JJ Ma JQ Cao WC Jin SG Study on the determinants regarding malaria epidemics in Anhui province during 2004-2006 Chinese Journal of Epidemiology 2009 30 1 38 41 (in Chinese) 19565846

[B15] Zhang W Wang L Fang L Ma J Xu Y Jiang J Hui F Wang J Liang S Yang H Spatial analysis of malaria in Anhui province, China Malar J 2008 7 206 10.1186/1475-2875-7-206 18847489 PMC2572066

[B16] Lines J Whitty CJ Hanson K Prospects for eradication and elimination of malaria: A technical briefing for DFID London School of Hygiene & Tropical Medicine 200722118883

[B17] Tanner M de Savigny D Malaria eradication back on the table Bull World Health Organ 2008 86 2 82 10.2471/BLT.07.050633 18297155 PMC2647379

[B18] Liu QY Liu XB Prevention and control of vector Anopheles: a key approach for malaria elimination in China Chin J Vector Biol & Control 2010 21 5 409 413 (in Chinese)

[B19] Qu FY Historical review on the classification and rectification of Anopheles anthropophagus to An. lesteri in China Chinese Journal of Parasitology and Parasitic Diseases 2008 26 3 234 235 19160973

[B20] Gu ZC Shang LY Chen JS Zheng X Su YJ Li AM Liu H Luo MZ Qian HL Tang LH The role of Anopheles anthropophagus in malaria transmission in in Xinyang City of Henan Province Chinese Journal of Parasitology and Parasitic Diseases 2001 19 4 221 224 12571970

[B21] Chow CY Malaria vectors in China Chinese Journal of Entomology 1991 Special Publ 6 67 79 (in Chinese)

[B22] Cox FE History of the discovery of the malaria parasites and their vectors Parasit Vectors 2010 3 1 5 10.1186/1756-3305-3-5 20205846 PMC2825508

[B23] Gou GX Li DF Shang LY Guo XS Wang WX Sui QL Shen YD Hao JL Hu ZT Liang DP The study on ecological habits of Anopheles sinensis in Guantang, Luyi county from 1971 to 1996 Chin J Vector Biol & Control 1998 9 133 134 (in Chinese)

[B24] Qu CZ Su TZ Vectorial capacity of malaria transmission of Anopheles sinensis in Zhengzhou in nature Joumal of Henan Medieal University 2000 35 394 396 (in Chinese)

[B25] Zhang HW Su YP Zhou GC Re-emerging malaria in Yongcheng city of Henan province Chin J Vector Biol & Control 2007 18 1 42 44 (in Chinese)

[B26] Zhou GC Zhang HW Su YP Evaluation of therapeutic measures of radical treatment for malaria in pre-transmission season in Yongcheng, Henan, China in 2007 Journal of Pathogen Biology 2009 4 2 112 114 (in Chinese)

[B27] Zhou GC Zhang HW Su YP Investigation into the results of control of malaria outbreak in Yongcheng County by biological control of mosquito larvae China Tropical Medicine 2009 9 2 228 229+259 (in Chinese)

[B28] Hu YX Miao YG Fan TB The further study on ecological habits of Anopheles sinensis in the area along Huang River and Huai River Chin J Parasit Parasitic Dis 1988 S1 135 (in Chinese)

[B29] Dia I Ba H Mohamed SA Diallo D Lo B Diallo M Distribution, host preference and infection rates of malaria vectors in Mauritania Parasit Vectors 2009 2 1 61 10.1186/1756-3305-2-61 19961573 PMC2791761

[B30] Jonathan FD Host-seeking strategies of mosquito disease vectors J Am Mosq Control Assoc 2005 21 sp1 17 22 10.2987/8756-971X(2005)21[17:HSOMDV]2.0.CO;2 16921679

[B31] Bowen MF The sensory physiology of host-seeking behavior in mosquitoes Annu Rev Entomol 1991 36 139 158 10.1146/annurev.en.36.010191.001035 1672499

[B32] Reisen WK Lothrop HD Meyer RP Time of host-seeking by Culex tarsalis (Diptera: Culicidae) in California J Med Entomol 1997 34 4 430 437 (438) 9220677 10.1093/jmedent/34.4.430

[B33] Laarman JJ The host-seeking behaviour of anopheline mosquitoes Trop Geogr Med 1958 10 4 293 305 13635845

[B34] Lardeux F Loayza P Bouchite B Chavez T Host choice and human blood index of Anopheles pseudopunctipennis in a village of the Andean valleys of Bolivia Malar J 2007 6 8 10.1186/1475-2875-6-8 17241459 PMC1783659

[B35] Schmidtmann ET Jones CJ Gollands B Comparative host-seeking activity of culicdides(Diptera:Ceratopogonidae) attracted to pastured livestock in central New York state, USA J Med Entomol 1980 17 3 221 231 (211)

[B36] Masuoka P Klein TA Kim HC Claborn DM Achee N Andre R Chamberlin J Small J Anyamba A Lee DK Modeling the distribution of Culex tritaeniorhynchus to predict Japanese encephalitis distribution in the Republic of Korea Geospat Health 2010 5 1 45 57 21080320 10.4081/gh.2010.186

[B37] Rosen L Shroyer DA Lien JC Transovarial transmission of Japanese encephalitis virus by Culex tritaeniorhynchus mosquitoes Am J Trop Med Hyg 1980 29 4 711 712 6250416 10.4269/ajtmh.1980.29.711

[B38] Rwegoshora RT Sharpe RG Baisley KJ Kittayapong P Biting behavior and seasonal variation in the abundance of Anopheles minimus species A and C in Thailand Southeast Asian J Trop Med Public Health 2002 33 4 694 701 12757212

[B39] Djenontin A Bio-Bangana S Moiroux N Henry MC Bousari O Chabi J Osse R Koudenoukpo S Corbel V Akogbeto M Culicidae diversity, malaria transmission and insecticide resistance alleles in malaria vectors in Ouidah-Kpomasse-Tori district from Benin (West Africa): A pre-intervention study Parasit Vectors 2010 3 83 10.1186/1756-3305-3-83 20819214 PMC2941749

[B40] Okumu FO Madumla EP John AN Lwetoijera DW Sumaye RD Attracting, trapping and killing disease-transmitting mosquitoes using odor-baited stations - The Ifakara Odor-Baited Stations Parasit Vectors 2010 3 12 10.1186/1756-3305-3-12 20193085 PMC2838860

[B41] Owino EA Trapping mosquitoes using milk products as odour baits in western Kenya Parasit Vectors 2010 3 55 10.1186/1756-3305-3-55 20573278 PMC2931484

[B42] Reddy MR Overgaard HJ Abaga S Reddy VP Caccone A Kiszewski AE Slotman MA Outdoor host seeking behaviour of Anopheles gambiae mosquitoes following initiation of malaria vector control on Bioko Island, Equatorial Guinea Malar J 2011 10 1 184 10.1186/1475-2875-10-184 21736750 PMC3146901

[B43] Forattini OP Kakitani I Massad E Marucci D Studies on mosquitoes (Diptera: Culicidae) and anthropic environment. 11--Biting activity and blood-seeking parity of Anopheles (Kerteszia) in south-eastern Brazil Rev Saude Publica 1996 30 2 107 114 10.1590/S0034-89101996000200001 9077008

[B44] Kweka EJ Mwang'onde BJ Mahande AM Optimization of odour-baited resting boxes for sampling malaria vector, Anopheles arabiensis Patton, in arid and highland areas of Africa Parasit Vectors 2010 3 75 10.1186/1756-3305-3-75 20723243 PMC2933686

[B45] Balenghien T Fouque F Sabatier P Bicout DJ Horse-, bird-, and human-seeking behavior and seasonal abundance of mosquitoes in a West Nile Virus focus of southern France J Med Entomol 2006 43 5 936 946 10.1603/0022-2585(2006)43[936:HBAHBA]2.0.CO;2 17017231

[B46] Burkett DA Lee WJ Lee KW Light, carbon dioxide, and octenol-baited mosquito trap and host-seeking activity evaluations for mosquitoes in a malarious area of the Republic of Korea J Am Mosq Control Assoc 2001 17 3 196 205 14529088

[B47] Kent RJ Molecular methods for arthropod bloodmeal identification and applications to ecological and vector-borne disease studies Mol Ecol Resour 2009 9 1 4 18 10.1111/j.1755-0998.2008.02469.x 21564560

[B48] Beier JC Perkins PV Wirtz RA Koros J Diggs D Gargan TP Koech DK Bloodmeal identification by direct enzyme-linked immunosorbent assay (ELISA), tested on Anopheles (Diptera: Culicidae) in Kenya J Med Entomol 1988 25 1 9 16 3357176 10.1093/jmedent/25.1.9

[B49] Washino RK Tempelis CH Mosquito host bloodmeal identification: methodology and data analysis Annu Rev Entomol 1983 28 179 201 10.1146/annurev.en.28.010183.001143 6131641

[B50] Lombardi S Esposito F Enzyme-linked immunosorbent assay (ELISA) for the identification of mosquito bloodmeals Parassitologia 1983 25 1 49 56 6543937

[B51] Tempelis CH Lofy MF A modified precipitin method for identification of mosquito bloodmeals Am J Trop Med Hyg 1963 12 825 831 14070778 10.4269/ajtmh.1963.12.825

[B52] Kent RJ Norris DE Identification of mammalian blood meals in mosquitoes by a multiplexed polymerase chain reaction targeting cytochrome B Am J Trop Med Hyg 2005 73 2 336 342 16103600 PMC4147110

[B53] Hatefi Y The mitochondrial electron transport and oxidative phosphorylation system Annu Rev Biochem 1985 54 1015 1069 10.1146/annurev.bi.54.070185.005055 2862839

[B54] Ngo KA Kramer LD Identification of mosquito bloodmeals using polymerase chain reaction (PCR) with order-specific primers J Med Entomol 2003 40 2 215 222 10.1603/0022-2585-40.2.215 12693851

[B55] Townzen JS Brower AV Judd DD Identification of mosquito bloodmeals using mitochondrial cytochrome oxidase subunit I and cytochrome b gene sequences Med Vet Entomol 2008 22 4 386 393 10.1111/j.1365-2915.2008.00760.x 19120966

[B56] Boakye DA Tang J Truc P Merriweather A Unnasch TR Identification of bloodmeals in haematophagous Diptera by cytochrome B heteroduplex analysis Med Vet Entomol 1999 13 3 282 287 10.1046/j.1365-2915.1999.00193.x 10514054

[B57] Lee JH Hassan H Hill G Cupp EW Higazi TB Mitchell CJ Godsey MS Unnasch TR Jr Identification of mosquito avian-derived blood meals by polymerase chain reaction-heteroduplex analysis Am J Trop Med Hyg 2002 66 5 599 604 12201598 10.4269/ajtmh.2002.66.599 PMC2586949

[B58] Fall AG Diaite A Lancelot R Tran A Soti V Etter E Konate L Faye O Bouyer J Feeding behaviour of potential vectors of West Nile virus in Senegal Parasit Vectors 2011 4 99 10.1186/1756-3305-4-99 21651763 PMC3118230

[B59] Zhou SS Wang Y Fang W Tang LH Malaria situation in the People's Republic of China in 2008 Chin J Parasit Parasitic Dis 2009 27 6 455 457 (in Chinese) 20232622

[B60] Diatta M Spiegel A Lochouarn L Fontenille D Similar feeding preferences of Anopheles gambiae and A. arabiensis in Senegal Trans R Soc Trop Med Hyg 1998 92 3 270 272 10.1016/S0035-9203(98)91005-7 9861393

[B61] Burkett DA Lee WJ Lee KW Kim HC Lee HI Lee JS Shin EH Wirtz RA Cho HW Claborn DM Late season commercial mosquito trap and host seeking activity evaluation against mosquitoes in a malarious area of the Republic of Korea Korean J Parasitol 2002 40 1 45 54 10.3347/kjp.2002.40.1.45 11949213 PMC2721055

[B62] Braack LE Coetzee M Hunt RH Biggs H Cornel A Gericke A Biting pattern and host-seeking behavior of Anopheles arabiensis (Diptera: Culicidae) in northeastern South Africa J Med Entomol 1994 31 3 333 339 8057306 10.1093/jmedent/31.3.333

[B63] Miller JE Gibson G Behavioral response of host-seeking mosquitoes (Diptera: Culicidae) to insecticide-impregnated bed netting: a new approach to insecticide bioassays J Med Entomol 1994 31 1 114 122 7908985 10.1093/jmedent/31.1.114

[B64] Muenworn V Sungvornyothin S Kongmee M Polsomboon S Bangs MJ Akrathanakul P Tanasinchayakul S Prabaripai A Chareonviriyaphap T Biting activity and host preference of the malaria vectors Anopheles maculatus and Anopheles sawadwongporni (Diptera: Culicidae) in Thailand J Vector Ecol 2009 34 1 62 69 20836806 10.1111/j.1948-7134.2009.00008.x

[B65] Lu BL Li PS Ji SH Fauna sinica insecta(Diptera: Culicidae) 1997 Beijing, China: Science Press (in Chinese)

[B66] Lee WJ Klein TA Kim HC Choi YM Yoon SH Chang KS Chong ST Lee IY Jones JW Jacobs JS Anopheles kleini, Anopheles pullus, and Anopheles sinensis: potential vectors of Plasmodium vivax in the Republic of Korea J Med Entomol 2007 44 6 1086 1090 10.1603/0022-2585(2007)44[1086:AKAPAA]2.0.CO;2 18047210

[B67] Collins FH Paskewitz SM A review of the use of ribosomal DNA (rDNA) to differentiate among cryptic Anopheles species Insect Molecular Biology 1996 5 1 1 9 10.1111/j.1365-2583.1996.tb00034.x 8630529

[B68] Ma YJ Qu FY Xu JN Zheng ZM Differentiation of Anopheles sinensis and Anopheles anthropophagus using a ribosomal DNA PCR assay Acad J Sec Mil Med Univ 1998 19 3 237 239 (in Chinese)

[B69] Porter CH Collins FH Species-diagnostic differences in a ribosomal DNA internal transcribed spacer from the sibling species Anopheles freeborni and Anopheles hermsi (Diptera:Culicidae) Am J Trop Med Hyg 1991 45 2 271 279 1877723 10.4269/ajtmh.1991.45.271

[B70] Rutledge CR Cornel AJ Meek CL Collins FH Validation of a ribosomal DNA-polymerase chain reaction species diagnostic assay for the common malaria mosquito (Diptera:Culicidae) sibling species complex J Med Entomol 1996 33 6 952 954 8961645 10.1093/jmedent/33.6.952

[B71] Kengne P Antonio-Nkondjio C Awono-Ambene HP Simard F Awolola TS Fontenille D Molecular differentiation of three closely related members of the mosquito species complex, Anopheles moucheti, by mitochondrial and ribosomal DNA polymorphism Med Vet Entomol 2007 21 2 177 182 10.1111/j.1365-2915.2007.00681.x 17550437

[B72] Ho C Chou TC Ch'En TH Hsueh AT The Anopheles hyrcanus group and its relation to malaria in east China Chin Med J 1962 81 71 78 13907849

[B73] Rueda LM Pecor JE Harrison BA Updated distribution records for Anopheles vagus (Diptera: Culicidae) in the Republic of Philippines, and considerations regarding its secondary vector roles in Southeast Asia Trop Biomed 2011 28 1 181 187 21602785

[B74] Lillie TH Kline DL Hall DW Host-seeking activity of Culicoides spp. (Diptera: Ceratopogonidae) near Yankeetown, Florida J Am Mosq Control Assoc 1988 4 4 485 493 3225567

[B75] Laarman JJ The host-seeking behaviour of the malaria mosquito Anopheles maculipennis atroparvus Acta Leiden 1955 25 1 144 13301352

[B76] Ghosh KK Das S Chakraborty S Bhattacharya S Hati AK Host preference of Anopheles annularis in different biotopes Indian J Malariol 1986 23 2 119 121 3569619

[B77] Suom C Ginsberg HS Bernick A Klein C Buckley PA Salvatore C Lebrun RA Host-seeking activity and avian host preferences of mosquitoes associated with West Nile virus transmission in the northeastern U.S.A J Vector Ecol 2010 35 1 69 74 10.1111/j.1948-7134.2010.00060.x 20618650

[B78] Grieco JP Achee NL Andre RG Roberts DR Host feeding preferences of Anopheles species collected by manual aspiration, mechanical aspiration, and from a vehicle-mounted trap in the Toledo District, Belize, Central America J Am Mosq Control Assoc 2002 18 4 307 315 12542188

[B79] Savage HM Anderson M Gordon E McMillen L Colton L Delorey M Sutherland G Aspen S Charnetzky D Burkhalter K Host-seeking heights, host-seeking activity patterns, and West Nile virus infection rates for members of the Culex pipiens complex at different habitat types within the hybrid zone, Shelby County, TN, 2002 (Diptera: Culicidae) J Med Entomol 2008 45 2 276 288 10.1603/0022-2585(2008)45[276:HHHAPA]2.0.CO;2 18402144

[B80] Loftin KM Byford RL Loftin MJ Craig ME Steiner RL Host preference of mosquitoes in Bernalillo County, New Mexico J Am Mosq Control Assoc 1997 13 1 71 75 9152878

[B81] Costantini C Sagnon NF della Torre A Diallo M Brady J Gibson G Coluzzi M Odor-mediated host preferences of West African mosquitoes, with particular reference to malaria vectors Am J Trop Med Hyg 1998 58 1 56 63 9452293 10.4269/ajtmh.1998.58.56

[B82] Mwandawiro C Boots M Tuno N Suwonkerd W Tsuda Y Takagi M Heterogeneity in the host preference of Japanese encephalitis vectors in Chiang Mai, northern Thailand Trans R Soc Trop Med Hyg 2000 94 3 238 242 10.1016/S0035-9203(00)90303-1 10974986

[B83] Harrison BA Scanlon JE The subgenus Anopheles in Thailand Contrib Am Entomol Inst 1975 12 1 307

[B84] Takken W The role of olfaction in host-seeking of mosquitoes:a review Insect Sci Appl 1991 12 287 295

[B85] Dekker T Takken W Braks MA Innate preference for host-odor blends modulates degree of anthropophagy of Anopheles gambiae sensu lato (Diptera: Culicidae) J Med Entomol 2001 38 6 868 871 10.1603/0022-2585-38.6.868 11761386

[B86] Pureswaran DS Poland TM Host selection and feeding preference of Agrilus planipennis (Coleoptera: Buprestidae) on ash (Fraxinus spp.) Environ Entomol 2009 38 3 757 765 10.1603/022.038.0328 19508785

[B87] Costantini C Gibson G Brady J Merzagora L Coluzzi M A new odour-baited trap to collect host-seeking mosquitoes Parassitologia 1993 35 1-3 5 9 7915027

[B88] Okumu FO Govella NJ Moore SJ Chitnis N Killeen GF Potential benefits, limitations and target product-profiles of odor-baited mosquito traps for malaria control in Africa PLoS One 2010 5 7 e11573 10.1371/journal.pone.0011573 20644731 PMC2904375

[B89] Sasa M Two years' observation on the seasonal activities and zoophilism of mosquitoes in Tokyo by animal trap method Jap J Exp Med 1951 20 509 517

[B90] Ree HI Hwang UW Lee IY Kim TU Daily survival and human blood index of Anopheles sinensis, the vector species of malaria in Korea J Am Mosq Control Assoc 2001 17 67 72 11345422

[B91] Ree HI Studies on Anopheles sinensis, the vector species of vivax malaria in Korea Korean J Parasitol 2005 43 3 75 92 10.3347/kjp.2005.43.3.75 16192749 PMC2712014

[B92] Boreham PF Lenahan JK Boulzaguet R Storey J Ashkar TS Nambiar R Matsushima T Studies on multiple feeding by Anopheles gambiae s.l. in a Sudan savanna area of north Nigeria Trans R Soc Trop Med Hyg 1979 73 4 418 423 10.1016/0035-9203(79)90167-6 555069

[B93] Scott TW Chow E Strickman D Kittayapong P Wirtz RA Lorenz LH Edman JD Blood-feeding patterns of Aedes aegypti (Diptera: Culicidae) collected in a rural Thai village J Med Entomol 1993 30 5 922 927 8254642 10.1093/jmedent/30.5.922

[B94] Klowden MJ Briegel H Mosquito gonotrophic cycle and multiple feeding potential: contrasts between Anopheles and Aedes (Diptera: Culicidae) J Med Entomol 1994 31 618 622 7932610 10.1093/jmedent/31.4.618

[B95] Wekesa JW Yuval B Washino RK Multiple blood feeding in Anopheles freeborni (Diptera: Culicidae) Am J Trop Med Hyg 1995 52 508 511 7611555 10.4269/ajtmh.1995.52.508

[B96] Anderson RA Brust RA Field evidence for multiple host contacts during blood feeding by Culex tarsalis, Cx. restuans, and Cx. nigripalpus (Diptera: Culicidae) J Med Entomol 1995 32 5 705 710 7473626 10.1093/jmedent/32.5.705

[B97] Koella JC Sorensen FL Anderson RA The malaria parasite, Plasmodium falciparum, increases the frequency of multiple feeding of its mosquito vector, Anopheles gambiae Proc R Soc Lond B Biol Sci 1998 265 763 768 10.1098/rspb.1998.0358 PMC1689045 9628035

[B98] Amerasinghe PH Amerasinghe FP Multiple host feeding in field populations of Anopheles culicifacies and An. subpictus in Sri Lanka Med Vet Entomol 1999 13 2 124 131 10.1046/j.1365-2915.1999.00160.x 10484158

[B99] Scott TW Amerasinghe PH Morrison AC Lorenz LH Clark GG Strickman D Kittayapong P Edman JD Longitudinal studies of Aedes aegypti (Diptera: Culicidae) in Thailand and Puerto Rico: blood feeding frequency J Med Entomol 2000 37 89 101 10.1603/0022-2585-37.1.89 15218911

[B100] Chow CY Note on the time of feeding of Anopheles hyrcanus sinensis and Anopheles mininus in the vicinity of Chungking Chin Med J 1949 67 489 490 (in Chinese)

[B101] Fredeen FJ Mason PG Meteorological factors influencing host-seeking activity of female Simulium luggeri (Diptera: Simuliidae) J Med Entomol 1991 28 6 831 840 1770519 10.1093/jmedent/28.6.831

[B102] Huang F Zhou S Zhang S Wang H Tang L Temporal correlation analysis between malaria and meteorological factors in Motuo County, Tibet Malar J 2011 10 54 10.1186/1475-2875-10-54 21375751 PMC3060153

[B103] Bassiouny HK Bioenvironmental and meteorological factors related to the persistence of malaria in Fayoum Governorate: a retrospective study East Mediterr Health J 2001 7 6 895 906 15332730

[B104] Ruiz MO Chaves LF Hamer GL Sun T Brown WM Walker ED Haramis L Goldberg TL Kitron UD Local impact of temperature and precipitation on West Nile virus infection in Culex species mosquitoes in northeast Illinois, USA Parasit Vectors 2010 3 1 19 10.1186/1756-3305-3-19 20302617 PMC2856545

[B105] Alemu A Abebe G Tsegaye W Golassa L Climatic variables and malaria transmission dynamics in Jimma town, South West Ethiopia Parasit Vectors 2011 4 30 10.1186/1756-3305-4-30 21366906 PMC3055844

[B106] Yang GJ Gao Q Zhou SS Malone JB McCarroll JC Tanner M Vounatsou P Bergquist R Utzinger J Zhou XN Mapping and predicting malaria transmission in the People's Republic of China, using integrated biology-driven and statistical models Geospat Health 2010 5 1 11 22 21080317 10.4081/gh.2010.183

[B107] Paaijmans KP Read AF Thomas MB Understanding the link between malaria risk and climate PNAS 2009 106 33 13844 13849 10.1073/pnas.0903423106 19666598 PMC2720408

[B108] Arunachalam N Samuel PP Hiriyan J Rajendran R Dash AP Short report: observations on the multiple feeding behavior of Culex tritaeniorhynchus (Diptera: culicidae), the vector of Japanese encephalitis in Kerala in southern India Am J Trop Med Hyg 2005 72 2 198 200 15741557

